# Visual-Somatosensory Integration and Quantitative Gait Performance in Aging

**DOI:** 10.3389/fnagi.2018.00377

**Published:** 2018-11-27

**Authors:** Jeannette R. Mahoney, Joe Verghese

**Affiliations:** ^1^Division of Cognitive and Motor Aging, Department of Neurology, Albert Einstein College of Medicine, Bronx, NY, United States; ^2^Division of Geriatrics, Department of Medicine, Albert Einstein College of Medicine, Bronx, NY, United States

**Keywords:** multisensory processing, sensorimotor integration, gait, falls, mobility

## Abstract

**Background:** The ability to integrate information across sensory modalities is an integral aspect of mobility. Yet, the association between visual-somatosensory (VS) integration and gait performance has not been well-established in aging.

**Methods:** A total of 333 healthy older adults (mean age 76.53 ± 6.22; 53% female) participated in a visual-somatosensory simple reaction time task and underwent quantitative gait assessment using an instrumented walkway. Magnitude of VS integration was assessed using probability models, and then categorized into four integration classifications (superior, good, poor, or deficient). Associations of VS integration with three independent gait factors (Pace, Rhythm, and Variability derived by factor analysis method) were tested at cross-section using linear regression analyses. Given overlaps in neural circuitry necessary for both multisensory integration and goal-directed locomotion, we hypothesized that VS integration would be significantly associated with pace but not rhythm which is a more automatic process controlled mainly through brainstem and spinal networks.

**Results:** In keeping with our hypothesis, magnitude of VS integration was a strong predictor of pace (β = 0.12, *p* < 0.05) but not rhythm (β = −0.01, *p* = 0.83) in fully-adjusted models. While there was a trend for the association of magnitude of VS integration with variability (β = −0.11, *p* = 0.051), *post-hoc* testing of individual gait variables that loaded highest on the variability factor revealed that stride length variability (β = −0.13, *p* = 0.03) and not swing time variability (β = −0.08, *p* = 0.15) was significantly associated with magnitude of VS integration. Of the cohort, 29% had superior, 26% had good, 29% had poor, and 16% had deficient VS integration effects.

**Conclusions:** Worse VS integration in aging is associated with worse spatial but not temporal aspects of gait performance.

## Introduction

Gait, a complex sensorimotor behavior involving coordination of neural networks, bones, muscles and joints, requires sensory information to aid in control of movement and to influence gait adaptation (Barbieri and Vitório, 2017). Effective integration of concurrent sensory stimulation is crucial for successful mobility. In our previous work, we demonstrate a protective effect of multisensory integration in aging whereby greater ability to integrate visual and somatosensory information was associated with increased balance performance, and decreased likelihood of falls (Mahoney et al., 2014, 2018).

To our knowledge, the association between visual-somatosensory (VS) integration and gait performance has not been established. Verghese and colleagues identified three independent gait domains using a factor analysis approach (Pace, Rhythm, and Variability; see Verghese et al., 2007b). The pace factor encompasses spatial parameters including gait speed, stride length, and percentage of gait cycle spent in double support (i.e., immobilized with two feet on the ground), whereas the rhythm factor includes temporal parameters such as cadence (number of steps per minute), swing time and stance time. The variability factor quantifies inconsistencies (measured in standard deviation units) of the highest loading gait variables of both spatial (stride length) and temporal (swing time) domains. Noteworthy, these gait domains have been verified by other investigators (Hollman et al., 2011; Lord et al., 2013; Verlinden et al., 2014).

Neuroimaging of the brain during walking has not been perfected yet; however existing models of locomotion reveal associations of neural activation in cortical (frontal/supplementary motor/parietal), subcortical (basal ganglia/thalamus), cerebellar, and brainstem regions with mobility outcomes (Holtzer et al., 2014). Noteworthy, multisensory integration effects have also been linked to cortical [frontal/motor/primary sensory areas/superior temporal sulcus (STS)] and subcortical (superior colliculus/thalamus) regions in cats, primates, and humans (Meredith and Stein, 1986; Stein et al., 2002; Calvert et al., 2004; Schroeder and Foxe, 2004). Given noticeable overlaps in the neural circuitry necessary for both sensory integration and goal-directed locomotion through space (sensory/motor regions, basal ganglia, and thalamus to name a few), we hypothesize that VS integration will be significantly associated with spatial aspects of gait (pace) and not with temporal aspects of gait (rhythm) which appear to be more automatic processes, influenced less by sensory inputs, and controlled mainly through brainstem and spinal networks (Kandel et al., 2013). The variability factor encompasses aspects of both pace and rhythm that could collectively be associated with VS integration; however, if our above hypothesis is supported, then VS integration should only be associated with stride length variability and not swing time variability.

## Material and methods

### Participants

Three-hundred-ninety-five participants enrolled in the Central Control of Mobility in Aging (CCMA) study at the Albert Einstein College of Medicine in Bronx, New York completed a multisensory simple reaction time (RT) experiment between June 2011 and June 2018. CCMA eligibility criteria required that participants be 65 years of age and older, reside in lower Westchester county, and speak English. Exclusion criteria for the CCMA included inability to independently ambulate, presence of dementia, significant bilateral vision, and/or hearing loss, active neurological or psychiatric disorders that would interfere with evaluations, recent or anticipated medical procedures that would affect mobility, and/or receiving hemodialysis treatment (see also Holtzer et al., 2013a,b). Presence of dementia was excluded using reliable cut scores from the AD8 Dementia Screening Interview (cutoff score ≥2; Galvin et al., 2005, 2006) and the Memory Impairment Screen (MIS; cutoff score < 5; Buschke et al., 1999); and later confirmed using consensus clinical case conference.

Additional exclusion criteria included history of severe unilateral vision (*n* = 5) and/or hearing loss (*n* = 4). All participants were required to successfully complete a sensory screening exam, where visual, auditory, and somatosensory acuity were formally tested to ensure appropriateness for the study. All CCMA participants were required to have bilateral visual acuity that was better or equal to 20/100 as measured by the Snellen eye chart. Individuals that were unable to hear a 2,000 Hz tone at 25 dB in both ears were not included in the CCMA study. As in our previous studies, presence or absence of neuropathy was diagnosed by the study clinician, and participants with severe neuropathy (unable to feel somatosensory stimulation) were not included (Mahoney et al., 2014, 2015, 2018). Additional exclusion criteria included inadequate multisensory performance (*n* = 40; see below) and prevalent dementia at study enrollment (*n* = 13).

After exclusions, the total study sample consisted of 333 older adults (mean age 76.53 ± 6.22 years; 53% female). All participants provided written informed consent to the experimental procedures, which were approved by the institutional review board of the Albert Einstein College of Medicine, Bronx, NY in accordance with the *Declaration of Helsinki*.

### Stimuli, task, and responses

Visual, somatosensory (vibratory pulses), and simultaneous VS stimuli were delivered through a custom-built stimulus generator (Zenometrics, LLC; Peekskill, NY, USA) that consisted of two control boxes, each housing a 15.88 cm diameter blue light emitting diodes (LEDs) and a 30.48 × 20.32 × 12.70 mm plastic housing containing a vibrator motor with 0.8 G vibration amplitude (Mahoney et al., 2015, 2018; Dumas et al., 2016). The devices were connected to a network control center, which allowed direct control for each device through the testing computer's parallel port. The devices were cycled on and off at precise predetermined intervals in any combination. A TTL (transistor-transistor-logic, 5 V, duration 100 ms) pulse was used to trigger the visual and somatosensory stimuli through E-Prime 2.0 software.

**Figure 1:**
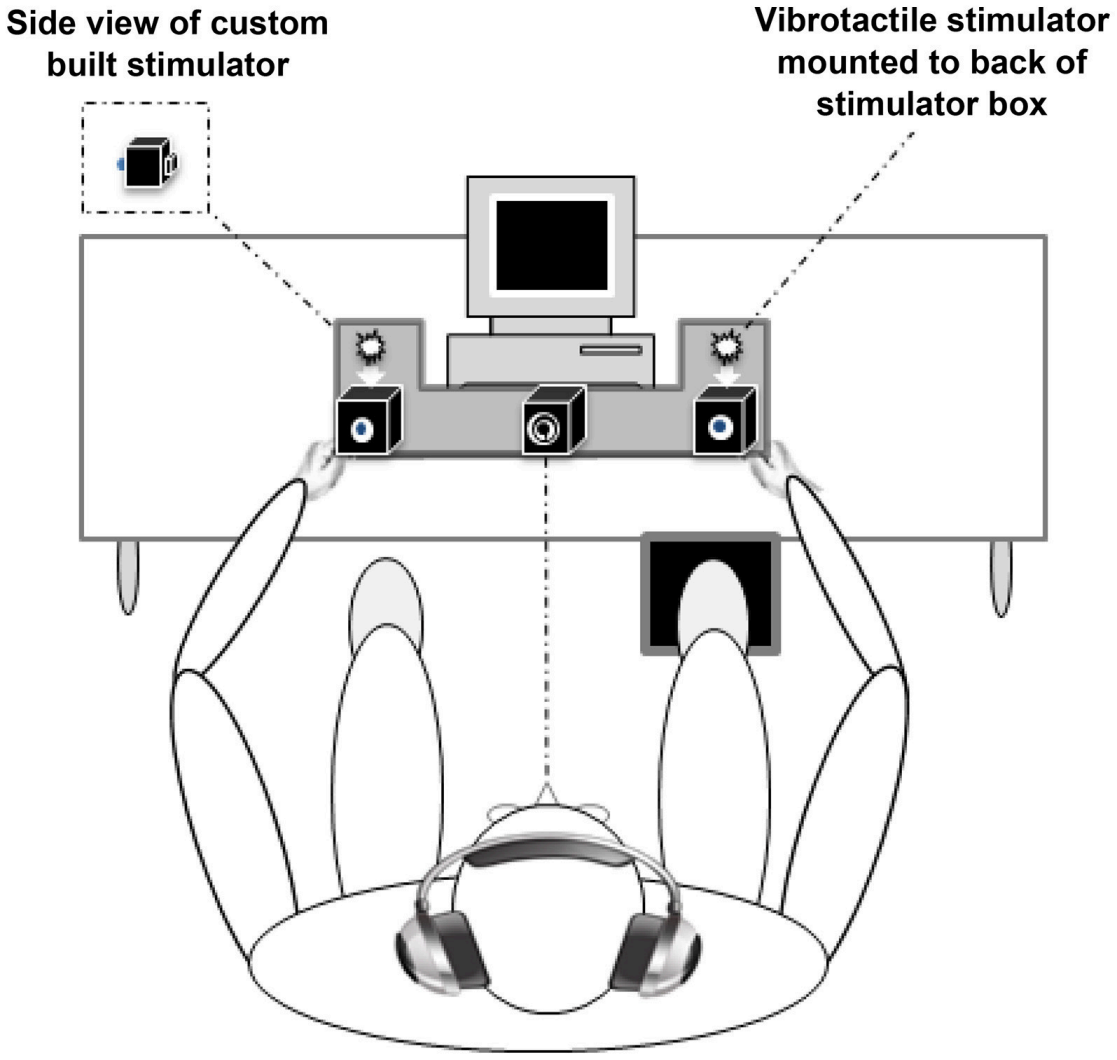
Experimental apparatus. Participants were required to make speeded responses to bilateral visual, somatosensory, and visual-somatosensory stimuli by pressing a foot pedal located under their right foot (Mahoney et al., 2015).

The control boxes were mounted to an experimental apparatus, which participants comfortably rested their hands upon, with their index fingers strategically placed over the vibratory motors on the back of the box and their thumb on the front of the box, under the LED (see Figure 1). A third dummy control box was placed in the center of the actual control boxes, at an equidistant length (28 cm) and contained a bull's eye sticker with a central circle of 0.4 cm diameter which served as the fixation point. To ensure that the somatosensory stimuli were inaudible, each participant was provided with headphones over which continuous white noise was played.

The three conditions were presented randomly with equal frequency and consisted of three blocks of 45 trials, for a total of 135 stimuli. Each block was separated by a 20 s break in order to reduce fatigue and facilitate concentration, and each subsequent block commenced immediately after the conclusion of the break. Participants were instructed to respond to all stimuli by pressing a stationary pedal located under their right foot as quickly as possible. Performance accuracy was defined as the number of accurate stimulus detections divided by 45 trials per condition. To prevent anticipatory effects, the inter-stimulus-interval varied randomly from 1 to 3 s. The duration of the entire experiment was approximately 7 min.

As in our previous multisensory studies, a 70% performance accuracy cutoff for all conditions was implemented to exclude participants with unreliable responses (*n* = 40; Mahoney et al., 2014, 2015, 2018). To be consistent with (Mahoney et al., 2018), data trimming procedures were purposefully avoided so as to not bias the distribution of the RT data (see Gondan and Minakata, 2016). If the participant failed to respond to any given stimulus, then that trial was considered inaccurate (omitted) and the corresponding RT was set to infinity rather than excluded from the analysis (Gondan and Minakata, 2016; Mahoney et al., 2018). To facilitate comparisons to other multisensory studies, the overall RT (average of all RTs regardless of condition) and overall RT facilitation effect (i.e., RT difference between the multisensory VS condition and the fastest unisensory condition) is included in Table 1.

**Table 1:** Demographic and clinical characteristics of study sample overall and by classification^*^.

**Variable**	**Overall cohort (*n* = 333)**	**Superior (*n* = 95)**	**Good (*n* = 87)**	**Poor (*n* = 96)**	**Deficient (*n* = 55)**	***p*-value ^∧^**
% Female	53	48	55	58	47	0.43
% Caucasian	77	83	72	73	80	0.19
% Moderate Visual Impairment	15	13	12	20	16	0.39
% with Neuropathy	6	6	5	5	7	0.91
Age (years)	76.53 (6.22) 65–93	76.02 (5.84) 65–92	75.72 (5.67) 66–91	76.98 (6.85) 65–93	77.93 (6.38) 67–92	0.15
Education (years)	15.02 (2.92) 5–21	15.14 (2.59) 8–21	15.10 (3.07) 5–21	15.08 (2.96) 8–21	14.54 (3.19) 7–21	0.62
GHS (0–10)	1.14 (0.96) 0–4	0.84 (0.83) 0–3	1.13 (0.96) 0–4	1.34 (0.94) 0–3	1.35 (1.09) 0–4	0.00
RBANS Total Score (65–135)	93.99 (11.59) 65–132	95.29 (11.65) 76–130	95.51 (11.11) 65–118	92.89 (10.90) 65–132	91.15 (12.94) 65–126	0.06
Overall RT (ms)	403.35 (118.36) 243–1,322	391.79 (91.47) 258–822	387.19 (79.74) 243–764	401.65 (116.39) 248–944	451.85 (185.87) 278–1,322	0.01
Somatosensory RT (ms)	441.53 (121.49) 252–1,228	429.97 (103.04) 273–880	429.52 (92.71) 271–743	437.67 (118.39) 252–905	487.23 (176.89) 289–1,228	0.02
Visual RT (ms)	404.95 (127.92) 233–1,425	395.95 (94.57) 262–805	387.58 (97.54) 233–1,050	404.58 (130.08) 250–1,019	448.58 (193.77) 275–1,425	0.04
Multisensory VS RT (ms)	364.37 (120.02) 213–1,318	349.84 (86.18) 239–781	346.44 (69.88) 226–617	362.71 (119.11) 213–914	420.74 (196.90) 245–1,318	0.00
RT facilitation time (ms)	30.36 (36.20) −135 to 148	38.07 (26.69) −45 to 148	33.88 (22.12) −24 to 84	26.71 (43.83) −135 to 132	17.83 (48.50) −123 to 112	0.08
VS integration ^#^	0.04 (0.14) −0.32 to 0.41	0.16 (0.09) 0.04–0.41	0.08 (0.08) −0.05 to 0.30	−0.05 (0.12) −0.31 to 0.26	−0.11 (0.08) −0.32 to 0.01	0.00
Velocity (cm/s)	100.35 (21.52) 49–167	105.46 (21.39) 55–167	102.60 (19.35) 60–150	96.60 (21.02) 49–143	94.47 (23.70) 52–153	0.00
Stride Length (cm)	117.07 (18.91) 66–165	122.06 (17.91) 70–158	118.62 (16.50) 72–165	114.47 (18.67) 66–155	110.57 (22.13) 70–162	0.00
Double Support %	31.50 (4.96) 18–48	30.52 (4.91) 18–48	30.59 (4.35) 22–45	32.44 (5.10) 21–48	32.99 (5.14) 23–47	0.00
Swing Time (s)	0.40 (0.04) 0.27–0.59	0.40 (0.05) 0.31–0.59	0.40 (0.04) 0.32–0.49	0.40 (0.04) 0.27–0.51	0.39 (0.05) 0.27–0.57	0.29
Cadence (steps/min)	102.84 (11.45) 69–148	103.76 (12.25) 69–148	103.99 (11.10) 83–132	101.13 (10.83) 73–130	102.43 (11.53) 69–127	0.30
Stance Time (s)	0.78 (0.11) 0.50–1.20	0.76 (0.10) 0.50–1.10	0.76 (0.10) 0.56–1.02	0.79 (0.11) 0.62–1.20	0.79 (0.12) 0.60–1.18	0.09
Stride Length Variability (SD)	3.70 (2.00) 0–13	3.53 (1.84) 1–13	3.32 (1.84) 0–8	3.99 (2.18) 1–13	4.07 (2.05) 1–10	0.05
Swing Time Variability (SD)	0.02 (0.01) 0.00–0.13	0.02 (0.02) 0.00–0.13	0.02 (0.01) 0.00–0.06	0.02 (0.01) 0.00–0.06	0.02 (0.02) 0.00–0.07	0.21

* Values are presented as mean ± SD for continuous variables and % for dichotomous variable.

# *Area under the curve of the CDF difference wave over the 0–10% percentile*.

∧ *Result of Between Groups One-Way ANOVAs*.

### Quantification of multisensory integration using the race model inequality

When two sources of sensory information are presented concurrently, they offer synergistic information that gives rise to faster responses, namely a redundant signals effect (Kinchla, 1974). Race models, commonly implemented to examine multisensory effects, are robust probability (P) models that compare the cumulative distribution function (CDF) of combined unisensory visual (V) and unisensory somatosensory (S) reaction times with an upper limit of one [min [P(RT_V_ ≤ *t*) + P(RT_S_ ≤ *t*), 1] to the CDF of multisensory VS reaction times [P(RT_VS_ ≤ *t*)] (Miller, 1982; Maris and Maris, 2003; Colonius and Diederich, 2006). For any latency *t*, the race model inequality (RMI) *holds* when the CDF of the **actual** multisensory condition [P(RT_VS_ ≤ *t)*] is less than or equal to the **predicted CDF** [min (P(RT_V_ ≤ *t*) + P(RT_S_ ≤ *t*), 1)]. Note that these CDFs take all RTs into account and have been extensively reviewed and utilized in our previous studies (Mahoney et al., 2015, 2018; Dumas et al., 2016). Acceptance of the above RMI suggests that unisensory signals are processed in parallel, such that the fastest unisensory signal could produce the actual response (i.e., the “winner” of the race). However, when the actual CDF is greater than the predicted CDF, the RMI is *rejected* and the RT facilitation is the result of multisensory interactions that allow signals from redundant information to integrate or combine non-linearly.

**Figure 2:**
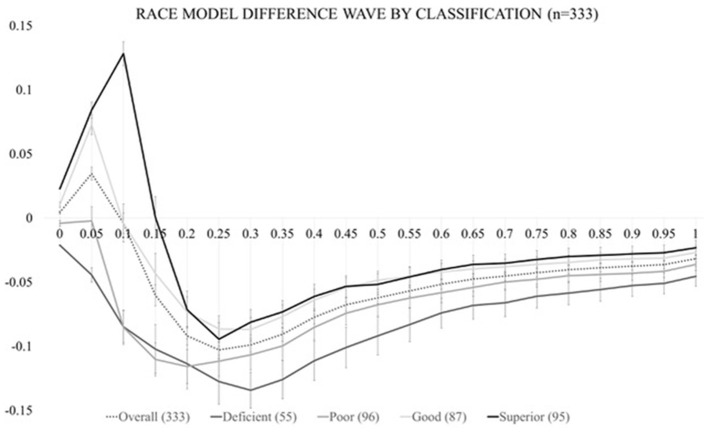
Test of the race model. The CDF difference waves over the trajectory of averaged responses for the entire study cohort (dashed trace) and for each of the four multisensory integration classifications (solid traces).

In order to calculate the race model violation, RTs must be sorted by condition in ascending order and the RT range across all stimulus (V, S, or VS) conditions must be calculated on an individual level. RT data are then quantized into 20 bins from the fastest RT (or zero percentile) to the slowest RT (hundredth percentile) in 5% increments (0%, 5%, …, 95%, 100%) separately for each condition. For example, let us suppose that for one individual the fastest RT was equal to 100 ms and the longest RT was equal to 1,000 ms (regardless of stimulus condition). Here, the single fastest RT of 100 ms would be represented at the 0th percentile. The next cumulative percentile bin (5%), would take all RTs that fell within the 100 ms + [5% of the range (1,000–100 ms = 900 ms range ^*^ 5% = 45 ms)] into account, so RTs between 100 and 145 ms. The 10% bin, would then consider RTs that were between 100 and 190 ms and so on until we reached the last RT (or 100%) percentile bin which would take all RTs from the 100–1,000 ms range into account. This method is implemented once for each of the three stimulus conditions and the probability of any RT occurring within each bin is calculated and transformed into a CDF. The CDF of the multisensory VS RTs represents the **actual** multisensory CDF, while the summation of the CDFs for both the visual and somatosensory CDFs (with an upper limit of 1) represents the **predicted** CDF. The difference in these two CDFs represents the Race Model inequality (RMI), where positive values are indicative of successful multisensory integration (also referred to as a violation of the race model). Figure 2 depicts the group-averaged difference between **actual** and **predicted** CDFs (dashed trace), where positive values (shaded area between 0 and 10th percentile) are indicative of VS integration (i.e., *rejected* RMI). The RMI was tested using Gondan's permutation test 2010 over the fastest 10% of responses and a robust violation was observed (*t*_max_ = 13.43, *t*_crit_ = 2.05, *p* < 0.001).

As in our previous study (Mahoney et al., 2018), actual CDF difference values for these three *violated* percentile bins (0, 5, and 10%) were used to (1) calculate the *area-under-the-curve* (AUC) which served as our independent variable of ‘magnitude of VS integration' for further statistical modeling and (2) determine VS integration classification. VS integration classification was assigned based on the number of violated percentile bins (0, 1, 2, or 3) during the 0–10th percentile. Classification definition was operationalized as follows: if all percentile values *violated* the RMI the individual was considered a “superior” integrator; if two values *violated* the RMI, the individual was considered a “good” integrator; if one value *violated* the RMI, the individual was considered a “poor” integrator; and if zero values *violated* the RMI, the person was considered a “deficient” integrator.” Figure 2 also depicts race model difference waves by integration classification (solid grayscale traces).

### Clinical evaluation

Global cognitive status was assessed using the Repeatable Battery for Assessment of Neuropsychological Status (Duff et al., 2008). As in our previous studies, global health scores (range 0–10) were obtained from dichotomous rating (presence or absence) of physician diagnosed diabetes, chronic heart failure, arthritis, hypertension, depression, stroke, Parkinson's disease, chronic obstructive pulmonary disease, angina, and myocardial infarction (Mahoney et al., 2011, 2014, 2015, 2018; Dumas et al., 2016).

### Gait evaluation

Quantitative gait assessments were conducted using a 28-foot instrumented walkway with embedded pressure sensors that provide various spatial and temporal gait parameters (GAITRite, CIR Systems, Havertown, PA). GAITRite, a valid system for measuring gait performance with excellent test-retest reliability (Bilney et al., 2003; Menz et al., 2004; Brach et al., 2008), is widely used in clinical and research settings (Verghese et al., 2007a). Here, steady-state locomotion was captured over a distance of 20 feet; data from the first and last 4 feet of the instrumented walkway (void of sensors) were purposefully excluded to eliminate initial acceleration and terminal deceleration. Participants were asked to walk on the mat at their “normal walking speed” in a quiet and well-lit room (see Verghese et al., 2002).

Similar to our previous work (Verghese et al., 2007b), factor analysis using the principal component method was performed on eight individual spatiotemporal gait parameters: gait velocity, stride length, percentage of double support, stride time, stance time, cadence, stride length variability, and swing time variability. The advantage of a factor approach using orthogonal varimax rotation is to reduce a large number of potentially correlated variables (while retaining most of the information) into a smaller number of uncorrelated independent factors that reduces the redundancy across individual variables. We identified a total of three independent gait factors (namely: Pace, Rhythm, and Variability) which later served as dependent variables in subsequent analyses. The pace factor includes three spatial variables: gait velocity, stride length, and percentage of immobilized gait or double support. The rhythm factor includes three temporal variables: stride time, stance time, and cadence which is number of steps per minute. The variability factor comprised both spatial (stride length variability) and temporal (swing time variability) facets of gait measured in SD units.

### Statistical analysis

Data were inspected descriptively and graphically and the normality of model assumptions was formally tested. Descriptive statistics (M ± SD) were calculated for continuous variables and between group ANOVAs were conducted. All data analyses were run using IBM's Statistical Package for the Social Sciences (SPSS), Version 24.

Three linear regression analyses (one for each gait factor) were performed with pace, rhythm, or variability serving as the dependent variable and VS integration as the independent variable in unadjusted models. Additional covariates were entered in a stepwise manner. In Step 2, age and gender were added as independent variables. In Step 3, additional independent variables included presence of moderate visual loss, presence of mild neuropathy, and global health score. If adjusted associations were significant, additional sensitivity analyses were conducted to determine whether adjustments for Overall RT or RBANS Total Index score impacted the association of VS integration with the dependent measure. In an effort to further scrutinize the variability factor, given our *a priori* hypotheses, two additional regression models were conducted to examine the individual association of VS integration with spatial (stride length variability) and temporal (swing time variability) variability components.

## Results

Demographic information is presented in Table 1 for both the overall cohort and for each multisensory classification group. Results demonstrate significant RMI violation over the fastest 10% of RTs using an established permutation test (Gondan, 2010); suggesting robust multisensory effects for the entire cohort. Difference values between actual and predicted CDFs were individually calculated for the *violated* percentile bins (0, 5, and 10%) and used to determine (1) multisensory integration classification group and (2) magnitude of VS integration. Based on our operational definition, our sample consisted of 95 superior integrators; 87 good integrators; 96 poor integrators; and 55 deficient integrators.

Factor analysis with varimax rotation yielded three orthogonal factors that accounted for over 87% of the variance in quantitative gait performance (Table 2). The factor with the highest variance, pace, had strong loadings by spatial parameters including velocity, stride length, and percent of gait cycle spent immobilized (double support). The second factor, rhythm, had strong loadings by temporal parameters including swing time, stance time, and cadence (or number of steps per minute). The last factor, variability, loaded highly on stride length (spatial), and swing time (temporal) variability measures. Mean factor score was 0 (SD 1), and factor scores can be conceptualized as summary risk scores with high scores representing worse performance.

**Table 2:** Factor loading of quantitative variables on three independent gait factors^#^.

**Gait variable**	**Pace**	**Rhythm**	**Variability**
Velocity (cm/s)	**0.876**	−0.409	−0.106
Stride length (cm)	**0.938**	0.080	−0.076
Double support %	−**0.897**	0.076	0.139
Swing time (s)	0.267	**0.933**	0.032
Cadence (steps/min)	0.346	−**0.919**	−0.100
Stance time (s)	−0.560	**0.783**	0.113
Stride length variability (SD)	−0.052	0.017	**0.950**
Swing time variability (SD)	−0.472	0.245	**0.494**
Variance explained %	39.78	32.09	15.08

# *Rotation method: Varimax with Kaiser Normalization*.

Bold values indicate loadings.

Results from the linear regression analyses (Tables 3A–E) reveal that VS integration processes, as quantified by the amount *area-under-the-curve* in the CDF difference wave, are associated with pace (β = 0.16, *p* ≤ 0.001) and variability (β = −0.12, *p* ≤ 0.04) factors, but not rhythm (β = 0.00, *p* = 1.00) in unadjusted models. The multisensory effect remained associated with pace even after controlling for age, gender, visual impairment, neuropathy, and global health score in models 2 through 3 (β = 0.12, *p* < 0.05), but not variability (β = −0.11, *p* = 0.051). Given our hypothesis regarding the association of VS integration with spatial and not temporal gait factors, we further examined the association of multisensory integration with stride length variability and swing time variability separately. Our findings revealed that only stride length variability (spatial aspect) was associated with VS integration in fully adjusted models (β = −0.13, *p* < 0.05). Sensitivity analyses further confirmed the significant association between VS integration and Pace even when adjusting for Overall RT (β = 0.13, *p* = 0.015) or RBANS Total index score (β = −0.13, *p* = 0.017). As well, the association between VS integration and Stride Length variability remained significant when adjusting for Overall RT (β = −0.12, *p* = 0.035) or RBANS Total index score (β = −0.13, *p* = 0.023).

**Table 3:** **(A–E)** Summary of linear regression models for predicting gait factors and/or variables.

**(A) Pace**		**Unstandardized coefficients**		**Standardized coefficients**	***t***	**Sig**.	**95% confidence interval for B**		**Collinearity statistics**	
		***B***	**Std. Error**	**Beta**			**Lower bound**	**Upper bound**	**Tolerance**	**VIF**
1	**VS integration**	1.17	0.40	0.16	2.96	0.00	0.39	1.95	1.00	1.00
2	**VS integration**	0.93	0.38	0.13	2.46	0.01	0.19	1.67	0.99	1.01
	Age	−0.05	0.01	−0.28	−5.45	0.00	−0.06	−0.03	0.99	1.01
	Gender	−0.28	0.10	−0.14	−2.70	0.01	−0.48	−0.08	1.00	1.00
3	**VS integration**	0.85	0.38	0.12	2.26	0.03	0.11	1.60	0.97	1.03
	Age	−0.05	0.01	−0.28	−5.44	0.00	−0.06	−0.03	0.99	1.02
	Gender	−0.28	0.10	−0.14	−2.69	0.01	−0.48	−0.08	0.98	1.02
	Visual Impairment	−0.14	0.15	−0.05	−0.99	0.32	−0.43	0.14	0.97	1.03
	Neuropathy	−0.33	0.23	−0.08	−1.47	0.14	−0.77	0.11	0.96	1.04
	GHS Score	−0.09	0.05	−0.08	−1.61	0.11	−0.19	0.02	0.97	1.03

**Table:** 

**Model summary**				
**Model**	***R***	**R square**	**Adjusted R square**	**Std. error of the estimate**
1	0.16	0.03	0.02	0.99
2	0.36	0.13	0.12	0.94
3	0.38	0.15	0.13	0.93

**Table:** 

**(B) Rhythm**		**Unstandardized coefficients**		**Standardized coefficients**	***t***	**Sig**.	**95% Confidence Interval for B**		**Collinearity statistics**	
		***B***	**Std. Error**	**Beta**			**Lower bound**	**Upper bound**	**Tolerance**	**VIF**
1	**VS integration**	0.00	0.40	0.00	0.00	1.00	−0.79	0.79	1.00	1.00
2	**VS integration**	−0.09	0.39	−0.01	−0.22	0.83	−0.85	0.68	0.99	1.01
	Age	−0.01	0.01	−0.03	−0.56	0.58	−0.02	0.01	0.99	1.01
	Gender	−0.51	0.11	−0.26	−4.78	0.00	−0.72	−0.30	1.00	1.00
3	**VS integration**	−0.08	0.39	−0.01	−0.21	0.83	−0.86	0.69	0.97	1.03
	Age	−0.01	0.01	−0.03	−0.52	0.60	−0.02	0.01	0.99	1.02
	Gender	−0.51	0.11	−0.26	−4.75	0.00	−0.72	−0.30	0.98	1.02
	Visual Impairment	−0.09	0.15	−0.03	−0.59	0.56	−0.39	0.21	0.97	1.03
	Neuropathy	−0.21	0.23	−0.05	−0.90	0.37	−0.67	0.25	0.96	1.04
	GHS Score	0.01	0.06	0.01	0.10	0.92	−0.11	0.12	0.97	1.03

**Table:** 

**Model summary**				
**Model**	***R***	**R square**	**Adjusted R square**	**Std. error of the estimate**
1	0.00	0.00	0.00	1.00
2	0.26	0.07	0.06	0.97
3	0.27	0.07	0.05	0.97

**Table:** 

**(C) Variability**		**Unstandardized coefficients**		**Standardized coefficients**	***t***	**Sig**.	**95% confidence interval for B**		**Collinearity statistics**	
		***B***	**Std. Error**	**Beta**			**Lower bound**	**Upper bound**	**Tolerance**	**VIF**
1	**VS integration**	−0.84	0.40	−0.12	−2.11	0.04	−1.62	0.06	1.00	1.00
2	**VS integration**	−0.78	0.40	−0.11	−1.96	0.05	−1.57	0.00	0.99	1.01
	Age	0.01	0.01	0.09	1.59	0.11	0.00	0.03	0.99	1.01
	Gender	−0.06	0.11	−0.03	−0.52	0.60	−0.27	0.16	1.00	1.00
3	**VS integration**	−0.79	0.40	−0.11	−1.96	0.05	−1.59	0.00	0.97	1.03
	Age	0.01	0.01	0.09	1.55	0.12	0.00	0.03	0.99	1.02
	Gender	−0.06	0.11	0.03	−0.52	0.60	−0.28	0.16	0.98	1.02
	Visual Impairment	0.09	0.16	0.03	0.58	0.56	−0.22	0.40	0.97	1.03
	Neuropathy	0.11	0.24	0.03	0.46	0.65	−0.36	0.58	0.96	1.04
	GHS Score	−0.02	0.06	0.02	−0.33	0.74	−0.13	0.10	0.97	1.03

**Table:** 

**Model summary**				
**Model**	***R***	**R square**	**Adjusted R square**	**Std. error of the estimate**
1	0.12	0.01	0.01	0.99
2	0.15	0.02	0.01	0.99
3	0.15	0.02	0.01	1.00

**Table:** 

**(D) Stride length variability**		**Unstandardized coefficients**		**Standardized coefficients**	***t***	**Sig**.	**95% confidence interval for B**		**Collinearity statistics**	
		***B***	**Std. Error**	**Beta**			**Lower bound**	**Upper bound**	**Tolerance**	**VIF**
1	**VS integration**	−1.93	0.79	−0.13	−2.44	0.02	−3.49	−0.37	1.00	1.00
2	**VS integration**	−1.83	0.80	−0.13	−2.30	0.02	−3.39	−0.26	0.99	1.01
	Age	0.03	0.02	0.09	1.59	0.11	−0.01	0.06	0.99	1.01
	Gender	−0.18	0.22	−0.04	−0.82	0.42	−0.60	0.25	1.00	1.00
3	**VS integration**	−1.81	0.80	−0.13	−2.25	0.03	−3.39	−0.23	0.97	1.03
	Age	0.03	0.02	0.09	1.54	0.12	−0.01	0.06	0.99	1.02
	Gender	−0.18	0.22	−0.05	−0.83	0.41	−0.61	0.25	0.98	1.02
	Visual impairment	0.20	0.31	0.04	0.66	0.51	−0.41	0.81	0.97	1.03
	Neuropathy	0.24	0.48	0.03	0.51	0.61	−0.70	1.18	0.96	1.04
	GHS score	0.00	0.12	0.00	0.01	1.00	−0.23	0.23	0.97	1.03

**Table:** 

**Model summary**				
**Model**	***R***	**R square**	**Adjusted R square**	**Std. error of the estimate**
1	0.13	0.02	0.02	1.98
2	0.16	0.03	0.02	1.98
3	0.17	0.03	0.01	1.98

**Table:** 

**(E) Swing Time Variability**		**Unstandardized coefficients**		**Standardized coefficients**	***t***	**Sig**.	**95% confidence interval for B**		**Collinearity statistics**	
		***B***	**Std. Error**	**Beta**			**Lower bound**	**Upper bound**	**Tolerance**	**VIF**
1	**VS integration**	−0.01	0.01	−0.10	−1.87	0.06	−0.02	0.00	1.00	1.00
2	**VS integration**	−0.01	0.01	−0.08	−1.48	0.14	−0.02	0.00	0.99	1.01
	Age	0.00	0.00	0.21	3.81	0.00	0.00	0.00	0.99	1.01
	Gender	0.00	0.00	0.05	0.90	0.37	0.00	0.00	1.00	1.00
3	**VS integration**	−0.01	0.01	−0.08	−1.46	0.15	−0.02	0.00	0.97	1.03
	Age	0.00	0.00	0.20	3.76	0.00	0.00	0.00	0.99	1.02
	Gender	0.00	0.00	0.05	0.90	0.37	0.00	0.00	0.98	1.02
	Visual Impairment	0.00	0.00	0.03	0.50	0.62	0.00	0.01	0.97	1.03
	Neuropathy	0.00	0.00	0.04	0.68	0.50	0.00	0.01	0.96	1.04
	GHS Score	0.00	0.00	0.00	−0.05	0.96	0.00	0.00	0.97	1.03

**Table:** 

**Model summary**				
**Model**	***R***	**R square**	**Adjusted R square**	**Std. error of the estimate**
1	0.10	0.01	0.01	0.01
2	0.24	0.06	0.05	0.01
3	0.24	0.06	0.04	0.01

## Discussion

The main objective of the current study was to determine whether ability to integrate concurrent VS information was associated with specific aspects of gait performance in older adults. Our findings reveal robust, but differential VS integration effects; 29% of the current study sample were superior VS integrators, while 26, 29, and 16% were considered good, poor, and deficient VS integrators, respectively. Our results demonstrate that magnitude of VS integration (i.e., *area-under-the-curve* in the CDF difference wave) was a strong predictor of spatial aspects of gait (i.e., pace factor). Magnitude of VS integration was not associated with temporal aspects of gait performance (rhythm), including swing time variability. The fact that magnitude of VS integration was however associated with stride length variability, does demonstrate a two-level dissociation between VS integration and spatial aspects of gait that is in keeping with our initial hypothesis.

In an effort to unpack the association of VS integration with spatial aspects of gait, we compared participants with poor or deficient multisensory integration abilities (*n* = 140) to those participants with superior or good multisensory integration abilities (*n* = 193). Results revealed that participants with good or superior VS integration maintained significantly faster gait velocity (103.55 vs. 95.93 cm/s; *p* = 0.001); longer strides (119.84 vs. 113.25 cm; *p* = 0.002); less percentage of gait cycle spent in double support (31 vs. 33%; *p* = 0.001) and less stride length variability (3.46 vs. 4.03 SD units: *p* = 0.01) compared to those with poor or deficient VS integration. While this information is helpful in characterizing the various spatial facets of gait, there are clear advantages to the application of a principal component approach when analyzing quantitative gait data.

Our finding that increased VS integration is linked to better goal-directed locomotion is directly in line with our hypothesis and likely a result of both processes activating similar neural circuitry. Multisensory integration effects have been linked to cortical [frontal/motor/primary sensory areas/superior temporal sulcus (STS)] and subcortical (superior colliculus/thalamus) regions in cats, primates, and humans (Meredith and Stein, 1986; Stein et al., 2002; Calvert et al., 2004). The lack of an association between VS integration and rhythm in our study could potentially be related to the fact that the temporal aspects of gait, emanating from brainstem and spinal networks, are less active during early, basic VS processing. While reports have indicated that multisensory inputs from brainstem can affect cortical integration processes, it is clear that the brainstem is primarily concerned with the temporal and spatial attributes of the sensory inputs, and thus the brainstem's role is more involved with modulation of information rather than information processing (Calvert et al., 2004).

Successful functioning and mobility in the real world rely on efficient multisensory integration processes that utilize feedback and feedforward neuronal loops between primary sensory, multisensory, and subcortical regions (see Calvert et al., 2004; Schroeder and Foxe, 2004; Meyer and Noppeney, 2011; Wallace, 2012). The thalamus plays an important role in the integration of sensory information, through cortico-cortical and cortical-subcortical transmissions (Sherman, 2005). Cortico-cortical and cortico-thalamic loops required for intact multisensory integration and mobility outcomes like balance and gait are notoriously compromised with aging. It is therefore logical that a disruption in shared neural circuitry, resulting from normal aging, disease, or any other potential variable, could adversely impact all processes relying on the functional and structural integrity of said circuit.

In an attempt to highlight the clinical significance of these findings, it should be noted that Verghese and colleagues posit that each 10 cm/s decrease in gait velocity is associated with a 7% increased risk for falls in our study populations (Verghese et al., 2009). The difference in gait velocity between the superior (105.46 cm/s) and deficient integrators (94.47 cm/s) was nearly 11 cm/s. Additionally, we recently revealed the clinical relevance of multisensory integration in aging in the context of balance and fall prediction (Mahoney et al., 2018) and our results indicate that older adults with superior VS integration abilities maintain: (1) better balance performance on the unipedal stance test (16.43 s) compared to deficient integrators (12.57 s) and (2) reduced occurrence of falls compared to deficient integrators for both prevalent (17 vs. 30%) and incident (42 vs. 80%) falls. Our initial studies highlight the significant association of VS integration (i.e., RT facilitation effect) with balance, falls, and physical activity level. However, the directionality of this association was seemingly paradoxical, where larger RT facilitation was associated with worse balance and increased falls (Mahoney et al., 2014, 2015). While a significant association between VS integration and balance and falls still remains (Mahoney et al., 2018), we posit that the directionally of this association is likely influenced by methodological modifications which included a new operational definition of VS integration based on magnitude of race model violation (not RT facilitation) and avoidance of data-trimming procedures that reportedly skew the CDF (Gondan and Minakata, 2016).

In terms of study limitations, a healthy young control group was purposefully excluded given known alterations in unisensory processing with increasing age. The ability to image the brain in motion is essential to determine the actual neural networks associated with the independent gait factors of pace, rhythm, and variability; hopefully continued advances in technology will afford the opportunity to launch this investigation sooner rather than later. Lastly, while overall cognitive functioning as measured by the RBANS was not significantly different between groups, it is possible that better VS integration is associated with better cognition, which could in turn influence the relationship of VS integration with spatial aspects of gait. Future studies should aim to determine the impact of cognition or cognitive status on the association of VS integration and various motor outcomes.

In conclusion, we provide support for the association of increased VS integration with increased gait performance, particularly with regard to spatial aspects of gait (pace) for older adults. Our main finding reveals that deficits in VS integration are linked to slower gait speed, shorter strides, and increased percentage of gait cycle spend immobilized with two feet on the ground (double support %). Additionally, worse VS integration was associated with increased stride length variability which has already been linked to increased fall-risk for older adults. Therefore, the current study continues to provide support for the notion that inefficient multisensory integration may be a potential novel mechanism for falls in older adults.

## Author contributions

JM: study concept and design, acquisition of participants and data, interpretation of data, grant support and manuscript preparation. JV: study concept and design, grant support, and preparation of manuscript.

### Conflict of interest statement

The authors declare that the research was conducted in the absence of any commercial or financial relationships that could be construed as a potential conflict of interest.

## References

[B1] Barbieri F. A. Vitório R. (2017). Locomotion and Posture in Older Adults: The Role of Aging and Movement Disorders. Cham: Springer International Publishing.

[B2] Bilney B. Morris M. Webster K. (2003). Concurrent related validity of the GAITRite walkway system for quantification of the spatial and temporal parameters of gait. Gait Posture 17, 68–74. 10.1016/S0966-6362(02)00053-X 12535728

[B3] Brach J. S. Perera S. Studenski S. Newman A. B. (2008). The reliability and validity of measures of gait variability in community-dwelling older adults. Arch. Phys. Med. Rehabil. 89, 2293–2296. 10.1016/j.apmr.2008.06.010 19061741 PMC2705958

[B4] Buschke H. Kuslansky G. Katz M. Stewart W. F. Sliwinski M. J. Eckholdt H. M.. (1999). Screening for dementia with the memory impairment screen. Neurology 52, 231–238. 9932936 10.1212/wnl.52.2.231

[B5] Calvert G. A. Spence C. Stein B. E. (2004). The Handbook of Multisensory Processes, eds. Cambridge, MA: The MIT Press.

[B6] Colonius H. Diederich A. (2006). The race model inequality: interpreting a geometric measure of the amount of violation. Psychol. Rev. 113, 148–154. 10.1037/0033-295X.113.1.148 16478305

[B7] Duff K. Humphreys Clark J. D. O'Bryant S. E. Mold J. W. Schiffer R. B. Sutker P. B. (2008). Utility of the RBANS in detecting cognitive impairment associated with Alzheimer's disease: sensitivity, specificity, and positive and negative predictive powers. Arch. Clin. Neuropsychol. 23, 603–612. 10.1016/j.acn.2008.06.004 18639437 PMC2570647

[B8] Dumas K. Holtzer R. Mahoney J. R. (2016). Visual-somatosensory integration in older adults: links to sensory functioning. Multisens. Res. 29, 397–420. 10.1163/22134808-00002521 29384609 PMC6198801

[B9] Galvin J. E. Roe C. M. Powlishta K. K. Coats M. A. Muich S. J. Grant E.. (2005). The AD8: a brief informant interview to detect dementia. Neurology 65, 559–564. 10.1212/01.wnl.0000172958.95282.2a 16116116

[B10] Galvin J. E. Roe C. M. Xiong C. Morris J. C. (2006). Validity and reliability of the AD8 informant interview in dementia. Neurology 67, 1942–1948. 10.1212/01.wnl.0000247042.15547.eb 17159098

[B11] Gondan M. (2010). A permutation test for the race model inequality. Behav. Res. Methods 42, 23–28. 10.3758/brm.42.1.23 20160282

[B12] Gondan M. Minakata K. (2016). A tutorial on testing the race model inequality. Atten. Percept. Psychophys. 78, 723–735. 10.3758/s13414-015-1018-y 26637234

[B13] Hollman J. H. McDade E. M. Petersen R. C. (2011). Normative spatiotemporal gait parameters in older adults. Gait Posture 34, 111–118. 10.1016/j.gaitpost.2011.03.024 21531139 PMC3104090

[B14] Holtzer R. Epstein N. Mahoney J. R. Izzetoglu M. Blumen H. M. (2014). Neuroimaging of mobility in aging: a targeted review. J. Gerontol. A Biol. Sci. Med. Sci. 69, 1375–1388. 10.1093/gerona/glu052 24739495 PMC4204614

[B15] Holtzer R. Mahoney J. Verghese J. (2013a). Intraindividual variability in executive functions but not speed of processing or conflict resolution predicts performance differences in gait speed in older adults. J. Gerontol. A Biol. Sci. Med. Sci. 69, 980–986. 10.1093/gerona/glt180 24285744 PMC4111295

[B16] Holtzer R. Wang C. Verghese J. (2013b). Performance variance on walking while talking tasks: theory, findings, and clinical implications. Age 36, 373–381. 10.1007/s11357-013-9570-7 23943111 PMC3889876

[B17] Kandel E. Schwartz J. Jessell T. Siegelbaum S. Hudspeth A. (2013). Principles of Neural Science, *eds* New York, NY: The McGraw-Hill Companies.

[B18] Kinchla R. (1974). Detecting target elements in multielement arrays: a confusability model. Percept. Psychophys. 15, 149–158.

[B19] Lord S. Galna B. Verghese J. Coleman S. Burn D. Rochester L. (2013). Independent domains of gait in older adults and associated motor and nonmotor attributes: validation of a factor analysis approach. J. Gerontol. A Biol. Sci. Med. Sci. 68, 820–827. 10.1093/gerona/gls255 23250001

[B20] Mahoney J. Cotton K. Verghese J. (2018). Multisensory integration predicts balance and falls in older adults. J. Gerontol. A Biol. Sci. Med. Sci. 10.1093/gerona/gly245 [Epub ahead of print]. 30357320 PMC6696711

[B21] Mahoney J. R. Dumas K. Holtzer R. (2015). visual-somatosensory integration is linked to physical activity level in older adults. Multisens. Res. 28, 11–29. 10.1163/22134808-00002470 26152050 PMC4830421

[B22] Mahoney J. R. Holtzer R. Verghese J. (2014). Visual-somatosensory integration and balance: evidence for psychophysical integrative differences in aging. Multisens. Res. 27, 17–42. 10.1163/22134808-00002444 25102664 PMC4280078

[B23] Mahoney J. R. Li P. C. Oh-Park M. Verghese J. Holtzer R. (2011). Multisensory integration across the senses in young and old adults. Brain Res. 1426, 43–53. 10.1016/j.brainres.2011.09.017 22024545 PMC3232048

[B24] Maris G. Maris E. (2003). Testing the race model inequality: a nonparametric approach. J. Math. Psychol. 47, 507–514. 10.1016/S0022-2496(03)00062-2

[B25] Menz H. B. Latt M. D. Tiedemann A. Mun San Kwan M. Lord S. R. (2004). Reliability of the GAITRite walkway system for the quantification of temporo-spatial parameters of gait in young and older people. Gait Posture 20, 20–25. 10.1016/S0966-6362(03)00068-7 15196515

[B26] Meredith M. A. Stein B. E. (1986). Visual, auditory, and somatosensory convergence on cells in superior colliculus results in multisensory integration. J. Neurophysiol. 56, 640–662. 3537225 10.1152/jn.1986.56.3.640

[B27] Meyer G. F. Noppeney U. (2011). Multisensory integration: from fundamental principles to translational research. Exp. Brain Res. 213, 163–166. 10.1007/s00221-011-2803-z 21800253

[B28] Miller J. (1982). Divided attention: evidence for coactivation with redundant signals. Cogn. Psychol. 14, 247–279. 7083803 10.1016/0010-0285(82)90010-x

[B29] Schroeder C. E. Foxe J. J. (2004). Multisensory convergence in early cortical processing, in The Handbook of Multisensory Processes, ed G. A. Calvert (Cambridge, MA: MIT Press), 295–309.

[B30] Sherman S. M. (2005). Thalamic relays and cortical functioning. Prog. Brain Res. 149, 107–126. 10.1016/s0079-6123(05)49009-3 16226580

[B31] Stein B. E. Wallace M. W. Stanford T. R. Jiang W. (2002). Cortex governs multisensory integration in the midbrain. Neuroscientist 8, 306–314. 10.1177/107385840200800406 12194499

[B32] Verghese J. Holtzer R. Lipton R. Wang C. (2009). Quantitative Gait Markers and Incident Fall Risk in Older Adults. J. Gerontol. A Biol. Sci. Med. Sci. 64, 896–901. 10.1093/gerona/glp033 19349593 PMC2709543

[B33] Verghese J. Kuslansky G. Holtzer R. Katz M. Xue X. Buschke H.. (2007a). Walking while talking: effect of task prioritization in the elderly. Arch. Phys. Med. Rehabil. 88, 50–53. 10.1016/j.apmr.2006.10.007 17207675 PMC1894901

[B34] Verghese J. Lipton R. B. Hall C. B. Kuslansky G. Katz M. J. Buschke H. (2002). Abnormality of gait as a predictor of non-Alzheimer's dementia. N. Engl. J. Med. 347, 1761–1768. 10.1056/NEJMoa020441 12456852

[B35] Verghese J. Wang C. Lipton R. B. Holtzer R. Xue X. (2007b). Quantitative gait dysfunction and risk of cognitive decline and dementia. J. Neurol. Neurosurg. Psychiatr. 78, 929–935. 10.1136/jnnp.2006.106914 17237140 PMC1995159

[B36] Verlinden V. J. van der Geest J. N. Hofman A. Ikram M. A. (2014). Cognition and gait show a distinct pattern of association in the general population. Alzheimers. Dement. 10, 328–335. 10.1016/j.jalz.2013.03.009 23849591

[B37] Wallace M. T. (2012). The Impact of multisensory alterations in human developmental disabilities and disease: the tip of the iceberg?, in The New Handbook of Multisensory Processing, ed B. E. Stein (Cambridge, MA: The MIT Press), 645–656.

